# Optically-Induced Cell Fusion on Cell Pairing Microstructures

**DOI:** 10.1038/srep22036

**Published:** 2016-02-25

**Authors:** Po-Fu Yang, Chih-Hung Wang, Gwo-Bin Lee

**Affiliations:** 1 Department of Power Mechanical Engineering, National Tsing Hua University, Hsinchu, Taiwan 30013; 2 Institute of Biomedical Engineering, National Tsing Hua University, Hsinchu, Taiwan 30013; 3 Institute of NanoEngineering and Microsystems, National Tsing Hua University, Hsinchu, Taiwan 30013

## Abstract

Cell fusion is a critical operation for numerous biomedical applications including cell reprogramming, hybridoma formation, cancer immunotherapy, and tissue regeneration. However, unstable cell contact and random cell pairings have limited efficiency and yields when utilizing traditional methods. Furthermore, it is challenging to selectively perform cell fusion within a group of cells. This study reports a new approach called optically-induced cell fusion (OICF), which integrates cell-pairing microstructures with an optically-induced, localized electrical field. By projecting light patterns onto a photoconductive film (hydrogen-rich, amorphous silicon) coated on an indium-tin-oxide (ITO) glass while an alternating current electrical field was applied between two such ITO glass slides, “virtual” electrodes could be generated that could selectively fuse pairing cells. At 10 kHz, a 57% cell paring rate and an 87% fusion efficiency were successfully achieved at a driving voltage of 20  V_pp_, suggesting that this new technology could be promising for selective cell fusion within a group of cells.

Cell fusion, whereby two or more cell types are merged into a hybrid cell, has been widely used for monoclonal antibody production, cell reprogramming, cancer immunotherapy, and tissue generation^1^,^2^,^3^,^4^. The hybrid cells can be generated from immunogenic, homogenic, or xenogeneic cell types that are fused in such a way as to yield hybrids of variable phenotypes. Cell fusion can be achieved by biological (e.g., virus-based)^5^, chemical (e.g., polyethylene glycol(PEG)-based)^6^,^7^, or physical (electrofusion) methods^8^,^9^. However, there are some limitations in the former, in particular. For instance, the fusion conditions need to be delicately regulated for different cell types, and it is not efficient for some kinds of cells. More seriously, biosafety is an issue with this approach. PEG-based methods are relatively simple and permit a variety of cell types to fuse^6^,^7^. With this approach, the hybrid cells are easy to isolate from the solution, and the procedure is relatively simple. However, the chemical methods also have some issues. For instance, it may take a longer period of time for cell fusion, and may cause permanent disruption of cell function of hybrid cells.

In addition to the aforementioned methods, another approach called electrofusion avoids several disadvantages of chemical and virus-based cell fusion approaches. With this approach, cells are exposed to a brief pulse of electricity in order to temporarily dilate and increase the permeability of their membranes^10^, thereby aiding in cell fusion. Specifically short-duration, high-voltage electrical pulses are applied to cause cell membrane fusion at the area of cell contact when sufficient transmembrane potential is induced. However, electrofusion usually requires a high-voltage power supply. Furthermore, for all three approaches, random cell pairing and unstable cell contact commonly occur. As a result, the efficiency and yield are seriously restricted when employing these traditional or benchtop methods.

Recently, several microfluidic devices have been demonstrated to alleviate the drawbacks of these traditional methods for cell fusion. For instance, dielectrophoresis (DEP) is a promising method for capturing cells and maintaining the integrity of cell pairings^11^,^12^,^13^. In the DEP procedure, as cell pairs are aggregated automatically on the microelectrodes, short-duration, and high-voltage electrical pulses are applied via the microelectrodes such that cell fusion is initiated. However, this method still faces the issue of random cell pairing. Alternatively, another DEP-based, cell fusion device that uses several lithography and lift-off processes to fabricate a micro-orifice array has recently been developed^14^,^15^. With this approach, different cell types could flow into the micro-orifices from different sides of the channel. Then, a DEP force was applied on the micro-orifices to trap cell pairs and induce cell fusion.

Another method that has been proven to pair cells with greater precision involves alternating the fluidic field^16^,^17^,^18^. In this approach, thousands of microstructures were fabricated within a microchannel for cell pairing. Cell-pairing dynamics were manipulated by controlling the flow field, and two cell types may be precisely paired in the same microstructure with pairing efficiencies up to 70%. Either PEG treatment or electrical pulses could be further applied to this microfluidic device for cell fusion, and 50% of the cell population has been found to be properly paired and fused over the entire device^16^. A similar microfluidic device which uses passive hydrodynamic forces and flow-induced cell deformation to trap different cell types within the same microstructure has been demonstrated^17^. As a result, a cell pairing rate as high as 80% (an average rate of around 70%) could be achieved. In this study, we adopted a similar microstructure-based technology that could automatically pair two cell types by manipulating flow fields. Note that the new cell-pairing microstructure is a one-layer structure containing two parts, which is different from the complicate multiple-layer structure reported in the previous studies.

There are still two problems associated with the microfluidic devices mentioned above. First, fixed microelectrodes require at least one metal micro-fabrication step. Moreover, it is not guaranteed that all cell pairs or cell contacts will experience the optimal electrical field strength for cell fusion. Recently, optically-induced dielectrophoresis (ODEP) systems or optoelectronic tweezers (OET)^19^ have been widely applied to manipulate dielectric and metallic particles^20^,^21^. Such optically-induced systems are constructed by illuminating light patterns onto photoconductive materials while an alternating-current (AC) electrical field is applied. Hydrogen-rich, amorphous silicon (a-Si: H) and photoconductive polymers^22^,^23^ are common materials used for this application. As light patterns are projected onto the photoconductive film, the conductivity of the illuminated region can be raised by several orders of magnitude, depending upon the illumination intensity, light wavelength, and photoconductivity of the material. Hence, the electrical field can be enhanced locally to form “virtual” electrodes when the top and bottom ITO glass substrates are subjected to an AC electric field. Therefore, metal microelectrodes fabricated by delicate photolithography processes can be replaced by the virtual electrodes. With this approach, dielectrophoresis for cell manipulation^19^, cell counting^24^, and electroporation^25^ have been successfully demonstrated. Since the virtual electrodes are generated by light patterns, the locally enhanced electrical field can be fine-tuned with ease by adjusting the projector position, line width, orientation, and colors of the light regime to each cell pair. The transmembrane potential of the cell membrane can then be induced such that cell fusion occurs when two cells are brought into contact. Therefore, there is greater flexibility for this new method, which could be operated at are relatively low voltage (typically a driving voltage of 20 V_pp_ with a frequency of 10 kHz). Herein we report a new approach called optically-induced cell fusion (OICF) in which integrated cell-pairing microstructures and an optically-induced, localized electrical field are utilized to achieve cell fusion with high pairing efficiency. There is the potential in this approach of adjusting the light pattern for each cell pair (which will vary slightly in size and location across the array) to physically place the electric field in the optimal place and intensity.

## Results

### Numerical simulation of transmembrane potential

To verify whether the optically-induced cell fusion system could generate appropriately sized minipores on the cell membranes, finite element analyses (FEA) software (COMSOL Multiphysics®, COMSOL Inc, USA) was used to simulate the transmembrane potential. Normally, a transmembrane potential from 0.5 to 1 V^26^,^27^,^28^ is appropriate for electroporation or cell electrofusion experiments. Transmembrane potential of a cell in an AC electrical field was modeled as follows^29^:


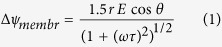


where 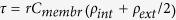, which is the relaxation time(s). *r, E,* and *ω* are the outer radius of cell, the magnitude of the AC electrical field (V/cm), and the angular frequency of the AC electrical field (rad/s), respectively. *C*_*membr*_ represents the capacitance of the membrane (F/cm^2^). *ρ*_*int*_ and *ρ*_*ext*_ (Ω·cm) represent the resistivity of the internal fluid and external media, respectively. Note that instead of using high-voltage DC electric pulses, an AC electrical field was applied on the OICF chip since not only does it induce dipoles on the membrane, but it also produces morphological changes on the membrane, which is useful for electroporation^26^.

**Figure 1:**
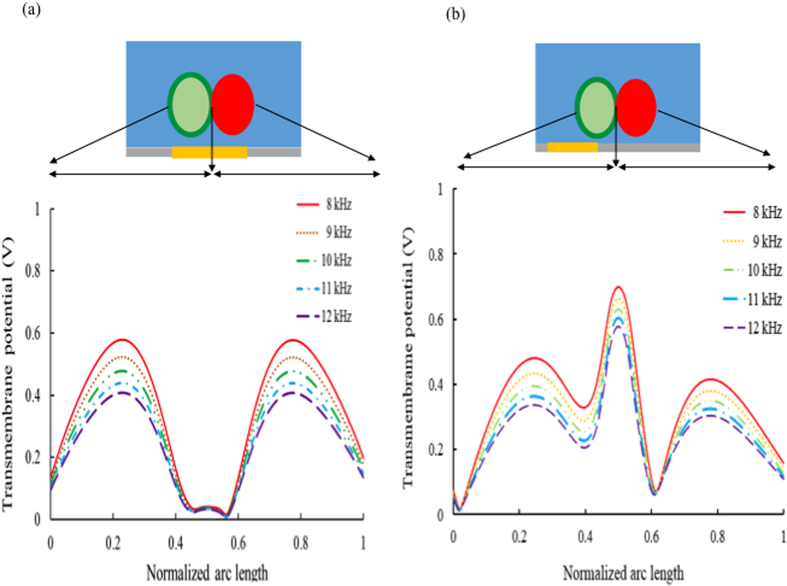
Numerical simulations of the transmembrane potential (20 Vpp). Different light pattern orientations have been modeled/simulated. (**a**) The light pattern was aligned towards the cell-cell interface. As the AC electrical field was applied, the maximum transmembrane potential occurred at the tops and bottoms of the cells. However, the transmembrane potential of the cell-cell contact area was not sufficient to induce cell fusion. (**b**) The light pattern was moved away from the cell at a distance of one cell radius. The electric field lines then passed through the cell contact area from the first cell to the second cell. Therefore, the maximum transmembrane potential was induced at the cell contact area. Maximum values ranging from 0.58 to 0.72 V were observed as the AC electric frequency was decreased from 12 to 8 kHz.

In this study, a two-dimensional (2D) OICF model was built (Fig. 1). Note that a three-dimensional (3D) model was first used to numerically simulate the electric field and transmembrane potential. The results showed that there was no significant difference between 2D and 3D models. The maximum value of the transmembrane potential occurs at the cell contact when the light pattern was moved away from the cell at a distance of one cell radius. Therefore, we decided to use the 2D model so that the numerical simulation could be performed more efficiently.

The heights of the liquid channel layer and the a-Si:H films were 30 and 1 μm, respectively. Cell diameter was set at 12 μm, and the width of the light pattern was chosen to be 30 μm. The light intensity was measured to be 1.86 W/cm^2^. By using the FEA software, the conditions (such as the line width, the spot position, the geometry of the light pattern, and the driving frequency of the AC electrical field) could be optimized prior to experimentation.

When the optical pattern was directly projected onto the pair of cells (Fig. 1a), two transmembrane potential peaks occurred on each cell. However, the transmembrane potential at the cell contact area was apparently not high enough to induce sufficient minipore formation at an applied voltage of 20 V_pp_. Furthermore, if the voltage or the light intensity was increased to enhance the transmembrane potential on the cell contact area, the transmembrane potential on other sides would also be increased, which might cause irreversible electrical breakdown of the cell membrane and consequent cell lysis. The main reason for the relatively low transmembrane potential at the cell contact area is because the electrical field lines did not pass through this area. Therefore, in this case, most of the electric field lines directly passed through each cell from the bottom to the top. As a result, the maximum transmembrane potential occurred at either the top or bottom of the cell. Therefore, it was not suitable to project the light pattern directly on the cell pair. In order to make the electric field lines pass through the cell contact, the virtual electrode (i.e. the light pattern) was moved away from the cell pair with a distance of a cell radius, and the numerical simulated results are shown in Fig. 1(b). It is clearly shown that under 20 V_pp_, the maximum value of the transmembrane potential occurs at the cell contact in this case, because the electric field lines pass through the cell contact, as expected. Moreover, the maximum value reached 0.58 to 0.72 V for different applied frequencies of the AC electric field (8–12 kHz), which is the appropriate magnitude to induce cell fusion. However, when the frequency was under 10 kHz, electrolysis occurred easily. From the above results, 10 kHz was therefore chosen as the operating frequency of the AC electric field.

### Cell pairing in microstructures

The new cell-pairing microstructure is a one-layer structure containing two parts. The upper part of the microstructure contains two pillars separated by a 15-μm gap that only permits one cell to pass through at any given time. The lower part contains two pillars with a 5-μm gap in between them so that the first cell is blocked while the second cell flows into the microstructure and is brought into contact with the first.

**Figure 2:**
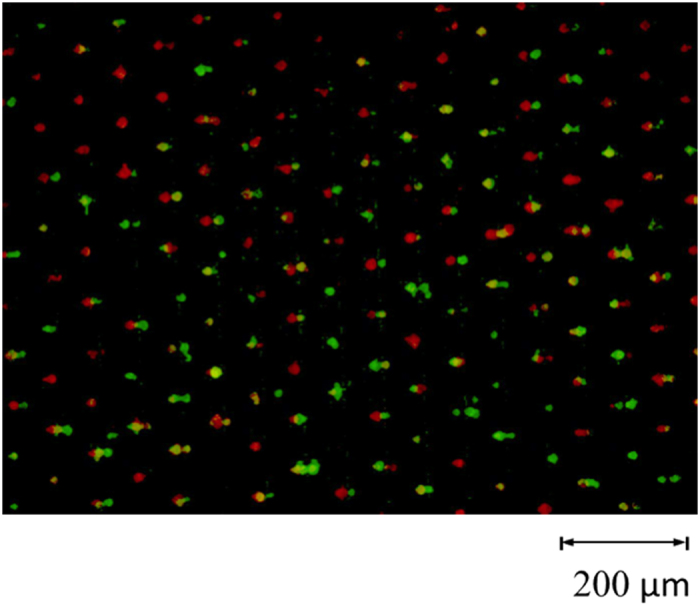
A representative fluorescent image of cell pairings in the cell-pairing microstructure array. The green and red spots represent HeLa and A549 cells, respectively. Merged images represent successful cell pairing events and, on average, a 57.14 ± 7.23% (n = 5 replicated experiments) cell-pairing rate was achieved.

As shown in Fig. 2, HeLa cells stained with a green reporter dye paired tightly with A549 cells stained with a red reporter dye within 15 min for 2400 cell pairs. Experimental results showed that a pairing rate of 57.17 ± 7.23% (standard deviation) was obtained after five replicate experiments, which was relatively higher than that achieved by random cell pairing.

### Cell fusion induced by virtual electrodes

**Figure 3:**
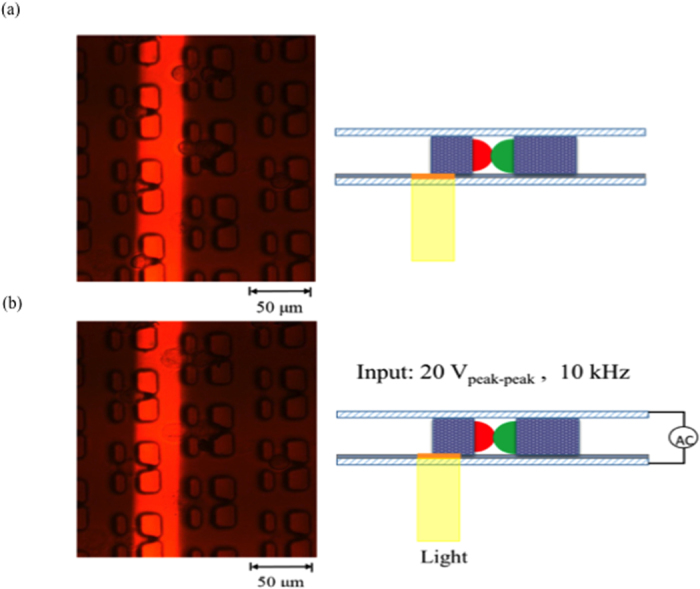
(**a**) A 30-μm light beam was projected on the desired position. (**b**) A driving AC voltage (20 Vpeak-peak, 10 kHz) was applied between the top and bottom ITO layers for 1s to generate the virtual electrode. The cell pairs began expansion, and the fusion process was subsequently induced.

**Figure 4:**
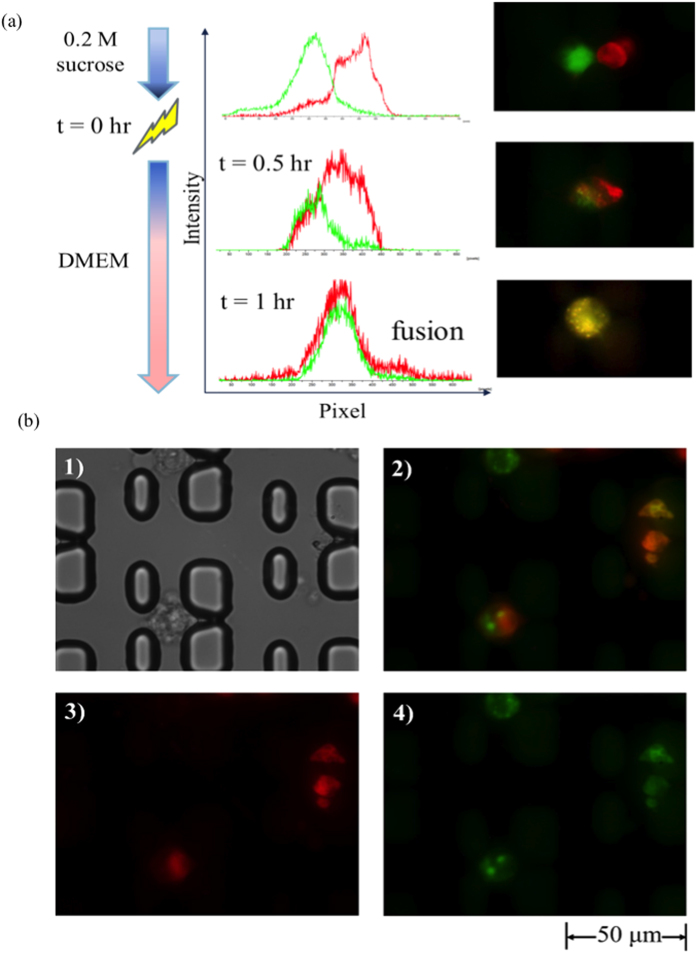
(**a**) Fluorescence intensity profile. After the virtual electrode was generated (t = 0 hr), two peaks of different fluorescence intensities(representing the two cells) were observed to move closer together, after a certain period of incubation (t = 1 hr), these peaks were observed to overlap, indicating successful cell fusion. (b-1) Microscopic image, (b-2) merged image and (b-3), (b-4) fluorescent images of the hybrid cell. The hybrid cells could emit both red (b-3) and green (b-4)fluorescence signals upon excitation under a fluorescence microscope, and the unmerged images have been shown.

After the cell-pairing process, 0.2 M sucrose was exchanged into the microchannel. The designed light pattern was then projected by a 20× condensing lens and a digital projector (PLC-XU106, SANYO Electric Co., Ltd, Japan) onto the position optimized by the numerical simulation model discussed above (as shown in Fig. 1). While an AC electrical field (20 V_p__p_,10 kHz) was applied between the two ITO layers, target cell pairs were observed to expand initially, and then the fusion process began (Fig. 3). The light pattern was then moved to the next row of microstructures to elicit fusion of cells in that row. Each row of microstructures was subjected to the applied electrical field for 1 s until all cell pairs had been exposed. Note that there are 160 rows in the microstructure array. Note that it requires 1s to expose light patterns for each row, and 4~5 s to align the light pattern to the suitable position on each row by moving the OICF chip on the microscopic stage (Fig. 4). Therefore, the total time required to expose all 2400 microstructures in the array was 15 min.

**Figure 5:**
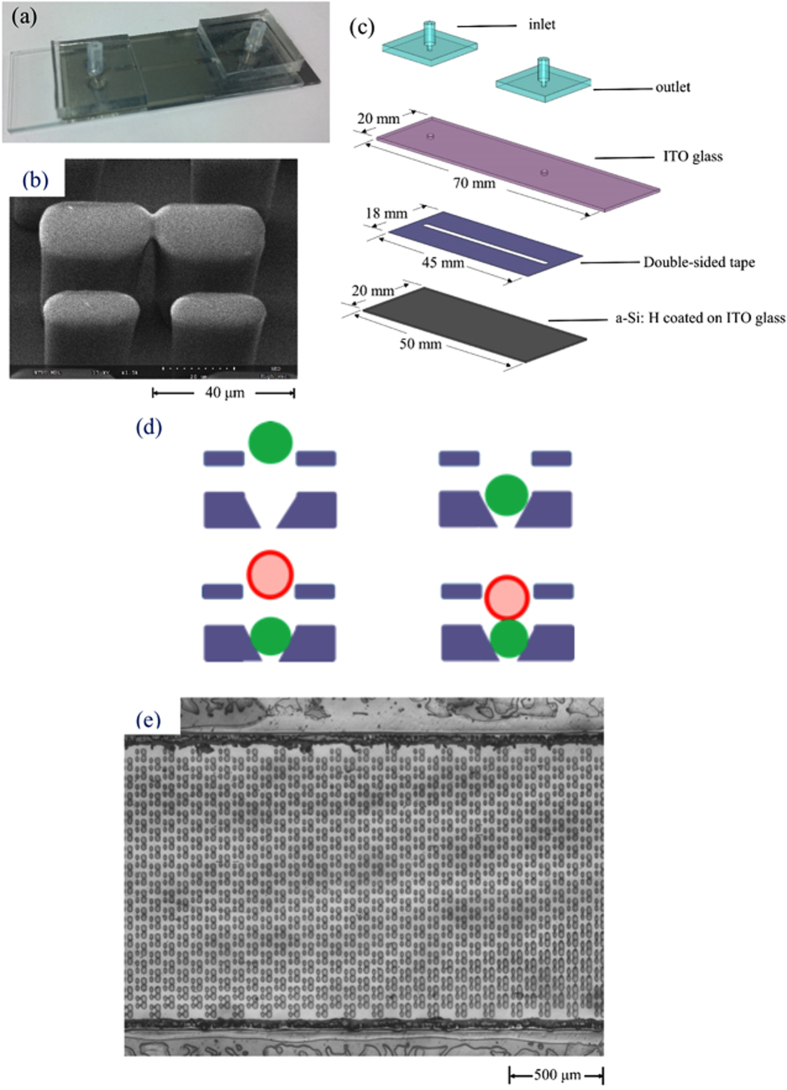
Images of the OICF chip. (**a**) A photograph of the OICF chip. (**b**) A scanning electron micrograph of the microstructures. (**c**) An exposed view of the OICF chip consisting of four layers. The first layer featured a PDMS inlet and a PDMS outlet bonded to an ITO glass. The bottom layer was an ITO glass slide coated with an a-Si: H layer. Two ITO glasses were bonded by a 30 μm-thick double-sided tape, and the microchannel was defined using a CO_2_ laser on the double-sided tape. The cell-pairing microstructures were fabricated on top of the a-Si: H film. (**d**) Design concept of the microstructures. The microstructures contain two parts. The upper part allows one cell to flow into one microstructure, and the 5 μm-gap in the lower part blocks the cell from moving further. Then, the second cell type enters and is paired with the first. (**e**) The top view of the microchannel. The dimensions of the OICF chip and the liquid microchannel were measured to be 80 × 20 × 10 mm(a) and 30 × 1.5 × 30 μm(a), respectively (length × width × height). In total, 2400 microstructures were fabricated on top of the a-Si: H film in the microchannel.

A fluorescence intensity profile was used to observe the cell fusion process (Fig. 5). Two peaks of different fluorescence intensities inside the two cells were clearly separated prior to cell fusion. These peaks moved closer to each other after the virtual electrode was generated. Finally, they were observed to fuse and overlap after 1 hour incubation, which means that the hybrid cell had been successfully formed. Note that only 1 s was used for initiating the cell fusion and 1 hour was used for cell incubation such that one may observe the fluorescent signals.

The fusion efficiency (hybrids/cell pairs) and the overall fusion efficiency (hybrids/microstructures) were further calculated. When using the OICF technique, 50 ± 9.42% (n = 5) overall fusion efficiency and 87.28 ± 6.94% (n = 5) fusion efficiency were achieved, which is much higher than that achieved by bench-top methods reported by the previous studies^26^,^27^,^28^. Table 1 shows the comparison of the OICF method, the PEG-based fusion performed on our cell-pairing microstructures, and benchtop methods using PEG and electrofusion^29^,^30^,^31^. From Table 1, the fusion efficiency of the OICF system is significantly higher than the other three treatment groups/methods. Furthermore, the cell-pairing microstructure also enhanced the efficiency at least 4-foldovertraditional benchtop methods. In summary, cell-pairing microstructures and the virtual electrode yielded the highest degree of cell fusion amongst the four methods tested herein with these two cell types.

## Discussion

We have constructed a convenient and flexible OICF microfluidic device that can perform cell fusion with high efficiency. The microstructures in the liquid channel are used to pair two different cell types at a rate of 57%. Note that the cell-pairing rate could be improved by optimizing the microstructures’ geometry or arrangement. Previous studies reported that 70%^16^ or 80%^17^ cell-pairing efficiencies could be achieved in their devices. However, the complicated multi-layer structures which require multiple lithography processes are inevitable. In this study, one-layer, one-lithography-process cell-pairing structure is used. Reasonable cell-paring rate is still achieved.

Furthermore, the virtual electrode was successfully projected into the expected region to induce an appropriate transmembrane potential at the cell-cell contact area. As a result, a cell fusion efficiency of 87% for cells paired on the microstructure could be achieved; this represents a significant improvement over benchtop methods, and even on-chip, PEG-based methods (44%)^29^,^30^,^31^. Additionally, overall cell fusion efficiency for OICF was measured to be 50%, which was significantly higher than values obtained with the benchtop PEG (<10%) and electrofusion (<20%) approaches. When compared with other microfluidic approaches^16^,^17^, we could comparable overall fusion efficiency in our device whilst using a lower voltage. In addition, the entire process only took only 90 minutes (including cell pairing and cell fusion), which was significantly shorter than the PEG-based approaches (>24 hours).

Our OICF chip could be a promising technique for dendritic-tumor cell fusion studies, which have made rapid advances in producing anti-tumor vaccines. Moreover, the virtual electrode could be established at any region on the photoconductive layer. Therefore, one could precisely choose which cell pairs to fuse while keeping the others intact, thus potentially serving as a promising and efficient technique for cancer immunotherapy, cell reprogramming, and other areas related to cellular biology that demand high-throughput, precise cell fusion in an arrayed fashion. Note that the re-suspended cells were stained with different fluorescence dyes. The entrance of the microstructure was designed to be only 25 μm (about 1.5–2 folds of the cell diameter). Hence, as the first cell type was injected into the microchannel, only one cell could flow into the microstructure at a time. As the first cell was trapped in the 5-μm gap, extra/unloaded cells/debris could be washed out with a high flow rate (20 μL/min, 1 × PBS). These extra cells could pass through the side gaps of the microstructure (15 μm, which is about the size of the cell diameter). Next, the second stained cells were injected into the OICF chip with a slower flow rate (10 μL/min) and flew into the microstructures. Finally, the second cell type could be paired with the first cell type. The dual mechanism and the size of microstructures could ensure the one-cell entry into the captured microstructure and then fused with another cell one by one. Therefore, the cell pairing was performed by the well-controlled flow velocity and the size-dependant microstructure. The cell pairing process is not a random process in this study. Furthermore, the cell viability was observed after 24 hr and was estimated to be around 70%.

## Methods

### Microfluidic chip

**Figure 6:**
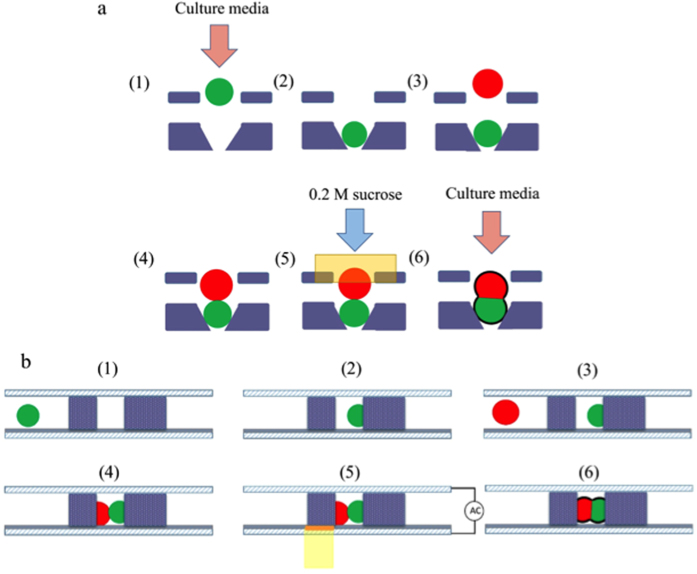
The working principle of OICF. (**a**) Top view and (**b**) cross-sectional view of the working illustration. (1) The first cell type in culture media is injected into the liquid channel and (2) blocked by the 5-μm gap in the cell-pairing microstructure. (3) The second cell type is then injected into the liquid channel and (4) paired with the first cell. (5) 0.2 M sucrose is exchanged into the liquid channel, and the virtual electrode is formed via illumination. (6) Finally, culture media was exchanged into the microchannel.

In order to perform cell fusion via virtual electrodes generated by illuminated light patterns, we first constructed a microfluidic chip that could both accommodate cell-pairing microstructures and form virtual electrodes (Fig. 6a). The chip was consisted of four layers (Fig. 6b); the first layer was made of poly dimethylsiloxane (PDMS) and functioned as the inlet and outlet of the liquid channel. The second layer was the ITO glass, which served as transparent top electrode. It also contained SU-8 microstructures for cell pairing. The PDMS inlet and outlet were bonded to the backside of the ITO glass via oxygen plasma treatment. The third layer was the liquid channel, which was made of a 30-μm-thick, double-sided tape. The microchannel was defined by a CO_2_ laser (VersaLASER VL-200, Universal Laser Systems, Inc., USA). The bottom layer was another ITO glass slide on which an a-Si: H film was coated. As light patterns are projected onto the a-Si: H film, electron-hole pairs are generated while the conductivity of the illuminated region is raised by several orders of magnitude. While an AC voltage is applied between the top and bottom ITO glass layers, the localized voltage drop is transferred to the liquid layer such that a non-uniform, locally enhanced, electrical field is produced. Then, the transmembrane potential on the cell membrane can be induced such that cell fusion occurs when two cells are brought into contact.

Initially, the fluid microchannel with the SU-8 cell-pairing microstructures was injected with 100 μL of poly(ethylene glycol)-block-poly (propylene glycol)-block-poly(ethylene glycol) (P123, Sigma-Aldrich, USA)solution for rinsing, which prevented bubble formation and cell adhesion. Phosphate-buffered saline (PBS) was then injected for washing out the residual P123, and Dulbecco’s modified eagle medium (DMEM) was then exchanged into the microchannel for subsequent incubation process.

Regarding the OICF procedure (Fig. 4). HeLa (cervical cancer cell line; described in more detail below) cells in culture media were loaded into the liquid channel with a syringe pump (KDS-270, Kd Scientific Inc., USA) at a constant flow rate of 20 μL/min for 5 min. The HeLa cells were then trapped by the SU-8 microstructures. Then, A549 (adenocarcinomic human alveolar basal epithelial cells; described in more detail below) cells were loaded into the same channel at a constant flow rate of 10 μL/min for 5 min with a syringe pump to be subsequently trapped by the cell-pairing microstructures. Afterwards, the two cell types were precisely paired in these microstructures. Finally, the culture media was exchanged at a flow rate of 20 μL/min with 0.2 M sucrose (Sigma-Aldrich, USA), which is a suitable buffer for OICF. Then, the optimized light patterns were projected onto the a-Si: H film with a digital projector (1.86 W/cm^2^, white light) while an AC electrical field was applied between the liquid channels. As a result, a locally enhanced electrical field was induced, which increased the permeability and/or the transmembrane potential of the cell membranes. When the transmembrane potential reached ~0.5–1V^26^,^27^,^28^, cytoplasm from opposite cell types could exchange through the minipores formed between the two cell types.

### Cells preparation

The HeLa cells (ATCC® CCL2™, USA) and A549 cells (ATCC CCL185™, USA) cultured under standard culture protocols were trypsinized and re-suspended in DMEM at a concentration of 10^5^ cells/mL. In order to observe the cell fusion process, HeLa and A549 cells were individually stained by two different kinds of fluorescent dyes (CellMask™ Green Plasma Membrane Stain and CellMask™ Deep Red Plasma membrane Stain, Molecular Probes®, Life Technologies™, USA, respectively) in accordance with the protocols provided by Life Technologies™.

### Experimental setup

**Figure 7:**
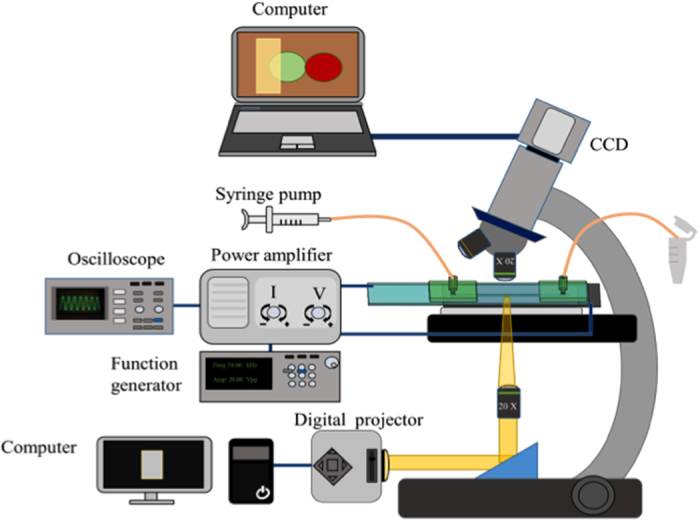
An illustration of the OICF system. A syringe pump was used for loading different cells into the microchannel. A function generator and a power amplifier supplied an appropriate electrical signal between the ITO layers, and the electrical signal was monitored by an oscilloscope. The optimized light regime was projected onto the photoconductive layer (a-Si: H) by a digital projector and a condensing lens. The pairing and fusion processes were observed under a florescent microscope equipped with a CCD.

The experimental setup of the OICF process is shown schematically in Fig. 7. The OICF chip was placed on an optical microscope (BX43, Olympus, Japan) to allow for *in-situ* observations by a charge-coupled device (CCD) camera (Evolution VF, Media Cybernetics, Inc., USA). The two different cell types were injected into the OICF chip with a syringe pump as described above and were paired in the microstructures. As described above, a digital projector illuminated a light pattern onto the a-Si: H film, thus creating a virtual electrode on the a-Si: H layer while an AC electrical field was applied between the top and bottom ITO layers. The AC signal was generated by a function generator (AFG-2125, Good Will Instrument Co., Ltd., Taiwan) and amplified (HA-405, PINTEK, Taiwan) to provide the required operating conditions (20 V_pp_, 10 kHz)for cell fusion. The output signal was monitored by a digital oscilloscope (GDS-1102-U, Good Will Instrument Co., Ltd.).

### PEG-based fusion on the microfluidic chip

PEG 6000 (Sigma-Aldrich Co. LLC., USA) was dissolved in glucose/KCI/NaCI/HCO_3_(GKN) buffer at a concentration of 50% (100 μL total volume). It was then used for cell fusion as a comparison for the developed method reported in the study. PEG solution was injected into the microchannel after the cell-pairing process and incubated for 30 min. Then, the cells were washed with 10× PBS at 5 μL/min for 15 min. DMEM was then injected into the microchannel at 10 μL/min for 10 min. Finally, the cells were incubated at 37 °C for an hour.

## Additional Information

**How to cite this article**: Yang, P.-F. *et al.* Optically-Induced Cell Fusion on Cell Pairing Microstructures. *Sci. Rep.* **6**, 22036; doi: 10.1038/srep22036 (2016).

## Figures and Tables

**Table 1:** Comparison of the OICF method developed herein, on-chip PEG fusion, and two traditional benchtop methods (PEG and electrofusion) on the cell pairing rate and fusion efficiency.

	Cell pairing rate (%) Paired cells/all microstructure	Fusion efficiency (%) Hybrids/paired cells	Overall efficiency (%) Hybrids/all microstructures
OICF on chip (HeLa & A549)	57.14 ± 7.23	87.28 ± 6.94*	50.08 ± 9.42
PEG on chip	53.08 ± 3.91	44.13 ± 10.21*	23.59 ± 6.64
PEG (bench)^26^	Not applicable	<10	<10
Electro fusion (bench)^27^	Not applicable	<20	<20

*p < 0.01, (two-tailed *t-*test).
